# Effects of an Alcohol-Related Harm Prevention Program among Out-of-School Female Adolescents

**DOI:** 10.3390/ijerph18084139

**Published:** 2021-04-14

**Authors:** Hyojin Park, Sungjae Kim, Jeongwoon Yang

**Affiliations:** 1 Salesio Girls’ High School, 21, Pilmun-daero 289 beon-gil, Dong-gu, Gwangju 61448, Korea; srmicaella@daum.net; 2 College of Nursing and the Research Institute of Nursing Science, Seoul National University, 103 Daehak-ro, Jongno-gu, Seoul 03080, Korea; 3 Department of Nursing, Kyungbok University, 425 Kyeongbokdae-ro, Namyangju-si 12051, Korea; jwyang120@gmail.com

**Keywords:** alcohol, prevention program, female, adolescents

## Abstract

This study aimed to evaluate the effects of an alcohol-related harm prevention program on out-of-school adolescent girls. This was a quasi-experimental study employing a randomized controlled non-synchronized design. There were 23 and 22 participants in the experimental and control groups, respectively. The program comprised three sessions aiming to motivate voluntary changes and promote autonomous decision-making. The experimental group showed significantly higher alcohol-related knowledge and substantially lower alcohol outcome expectancy than the control group. No significant differences emerged from drinking refusal, self-efficacy, or alcohol abstinence intention. This program could improve alcohol-related knowledge and reduce out-of-school adolescent girls’ positive alcohol outcome expectancy.

## 1. Introduction

According to the Korea Health Statistics [1], the percentage of high-risk drinkers in South Korea has been consistently rising every year since 2013 and reached 14.2% in 2017. Drinking problems among adolescent girls have been exacerbated in the recent past. Among female middle and high school students who drink alcohol, 49.9% showed a risk of problematic drinking behaviors, which indicated a moderate or higher than average amount of drinking per seating in the past 30 days (male: ≥5 shots of soju, female: ≥3 shots of soju; soju is a Korean vodka). Furthermore, 40.8% showed problematic drinking behaviors, which indicated experiencing two or more problematic behaviors after drinking in the past 12 months, as they initiated drinking by as early as 13 years old [1]. The fact that the age of drinking initiation decreased as the high-risk drinking rate and binge drinking rate increased showed that adolescent girls were heavily exposed to alcohol consumption and thus are highly vulnerable to drinking-related harm. Moreover, the rate of alcohol consumption among out-of-school adolescents, that is, those who are not affiliated with a school and have a history of running away from home or engaging in delinquency, was markedly higher (83.1%) than that among in-school adolescents (16.9%) [2]. This highlights the need for greater attention to the drinking habits of out-of-school adolescent girls as well as their protection.

Among adolescent girls, drinking leads to physical, psychological, and social problems [3] and is linked to drinking during pregnancy, thereby impacting childbirth in the future [4]. Alcohol hinders normal growth as it is toxic for adolescent brains, which are undergoing active structural and functional changes [3,5]. Furthermore, drinking undermines their judgment-making capacity and increases compulsivity [6], which, in turn, increases involvement in risky sexual activities and exposure to social risks, such as violence, sexual harassment, sexual violence, and dating violence. It also exposes them to the risk of unwanted pregnancy and sexually transmitted diseases [7]. The younger the age of drinking initiation, the greater the abuse of addictive substances such as narcotics. Habitual drinking in adolescent girls has the potential to lead to alcohol abuse and dependence in adulthood. If this habitual drinking remains unchanged and drinking behavior continues during pregnancy, it can lead to an irreversible condition known as fetal alcohol syndrome [7,8]. While these situations seem non-problematic during adolescence, it could become a serious problem in adulthood when individuals trying to become healthy mothers have difficulties ceasing alcohol use before and during pregnancy. It is important to provide interventions to impart accurate knowledge about alcohol among adolescents and help establish a healthy attitude toward it. As adolescents become fertile and begin to drink at this age, it is necessary to prevent alcohol use or to delay alcohol initiation as much as possible [9] to reduce drinking-related harm brought about by habitual drinking in adolescent girls who already drink [6]. Providing drinking prevention programs for female adults or pregnant women is also important.

In South Korea, drinking prevention interventions are primarily based in schools [10,11] and are run by school nurses or counselors. Hence, out-of-school adolescent girls cannot access these interventions after initiating alcohol use despite being at high risk of alcohol abuse because of continuous exposure to drinking environments. Adolescents’ drinking behaviors and drinking intentions are influenced by their drinking-related knowledge and attitude toward drinking [11]. This highlights the urgency of providing drinking prevention programs for out-of-school youth, especially for females, who are vulnerable to practical and potential drinking problems. Interventions for out-of-school adolescent girls who often run away from home and engage in delinquency should focus on the practical problems that they may experience in addition to providing information about drinking, and they should encourage autonomous motivation for alcohol abstinence.

Thus, this study aimed to investigate the effects of an alcohol-related harm prevention program focused on the problems caused by drinking in out-of-school female adolescents. This study specifically aimed to assess the effects of the program on alcohol-related knowledge, alcohol outcome expectancy, drinking refusal self-efficacy, and alcohol abstinence intention in out-of-school female adolescents. The study hypotheses were as follows:

**Hypothesis** **1** **(H1):** *The experimental group that participated in an alcohol-related harm prevention program will show a greater increase in their alcohol-related knowledge scores compared to the control group*.

**Hypothesis** **2** **(H2):** *The experimental group that participated in an alcohol-related harm prevention program will show a greater drop in their alcohol outcome expectancy scores compared to the control group*.

**Hypothesis** **3** **(H3):** *The experimental group that participated in an alcohol-related harm prevention program will show a greater increase in their drinking refusal self-efficacy scores compared to the control group*.

**Hypothesis** **4** **(H4):** *The experimental group that participated in an alcohol-related harm prevention program will show a greater increase in their alcohol abstinence intention scores compared to the control group*.

### 1.1. Theoretical Framework

**Figure 1:**
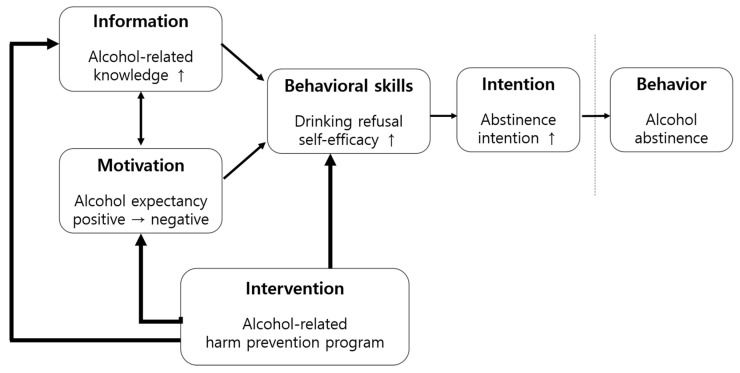
Conceptual framework of the study.

The theoretical framework of this study was established by integrating Ajzen and Madden’s [12] theory of planned behavior (TPB) with the information–motivation–behavioral skills (IMB) theory by Fisher and Fisher [13]. The IMB model posits that behavioral changes occur and that the changed behaviors are retained when an individual has acquired adequate information about the new behavior, is motivated to change the old behavior, and has the skills to engage in the newly acquired behavior [14]. The TPB states that behavioral intention is the direct determinant of switching to healthy behavior and that individuals with strong intentions make efforts to achieve a goal and are more easily motivated to change their behavior. Behavioral intention is influenced by the attitude based on beliefs about behavior, social norms, and perceived behavioral control. In this study, the TPB was integrated with the IMB model to set alcohol abstinence intention as a predictor of a change in behavior (alcohol abstinence) in the future. As the participants were to abstain from alcohol consumption during the six months they resided in the center, we predicted their alcohol abstinence in the future based on their alcohol abstinence intention instead of directly using the change in drinking behavior. To increase the alcohol abstinence intention, we hypothesized that individuals needed to have an increased self-efficacy to refuse to drink and that they would be motivated to change their drinking behaviors when they were provided with alcohol-related information leading to a change in their alcohol outcome expectancy. Thus, this study set alcohol-related knowledge, alcohol outcome expectancy, drinking refusal self-efficacy, and alcohol abstinence intention as the dependent variables and the alcohol-related harm prevention program as the independent variable to ultimately achieve alcohol abstinence or moderate drinking in out-of-school female adolescents (Figure 1).

### 1.2. Terminology

#### 1.2.1. Out-of-School Female Adolescents

In South Korea, out-of-school female adolescents refer to those under the age of 19 years who are not affiliated with an elementary, middle, or high school as per Article 2 of the Elementary of Secondary Education Act, or with any school that provides an equivalent curriculum [15]. In this study, out-of-school female adolescents referred to female adolescents under the age of 19 years who resided in a protection and treatment center known as the M Center. The M Center is an institution that provided social adaptation education instead of detaining youth under the age of 19.

#### 1.2.2. Alcohol-Related Harm Prevention Program

An alcohol-related harm prevention program focuses on preventing the adverse outcomes of drinking by delaying the age of alcohol initiation or delaying an increase in drinking frequency or amount of drinking [11,16]. The program used in this study was designed to increase alcohol-related knowledge, convert alcohol outcome expectancy, increase self-efficacy to refuse drinking in triggering situations, and elevate alcohol abstinence intention with the help of group lectures focused on female drinking problems and six sessions of small group activities to improve self-efficacy for out-of-school female adolescents who either had initiated alcohol use or were at high risk of initiating it.

#### 1.2.3. Alcohol Outcome Expectancy

The expectation of alcohol results means that girls (youth outside of school) feel that their identity and sense of belonging are established only through drinking in a peer group. Additionally, the temporary relief of emotional tension that alcohol consumption brings is referred to as the alcohol outcome expectancy.

## 2. Materials and Methods

### 2.1. Design

A non-equivalent control group pretest–post-test non-synchronized design was used to investigate the effects of an alcohol-related harm prevention program in out-of-school female adolescents (Table 1).

### 2.2. Participants

The participants of this study were out-of-school female adolescents residing in the M Center in Seoul, South Korea, whose caregivers provided written informed consent. The inclusion criteria were as follows: (a) female adolescents between the ages of 10 and 19 years who were eligible to be admitted to the facility and (b) those who did not attend a school at the time of study due to dropping out or being expelled from school. The exclusion criteria were as follows: (a) individuals who had resided in the facility for less than one month and (b) individuals scheduled to be discharged from the facility within two weeks. This was to include individuals who have stably adjusted to the facility environment.

The sample size was determined with reference to Cohen’s guidelines; for a two-group comparison with an alpha of 0.05, power of 0.80, and effect size of 0.50, the minimum sample size for each group was determined to be 17. Anticipating a 30% withdrawal rate, we set the size to 23 participants for each group. After explaining the study to 53 center residents, a list of 46 residents, whose caregivers provided consent and who met the inclusion criteria, was provided by the center. The researcher assigned a number to each participant and allocated them into the experimental and control groups based on the order generated by a computerized randomization software. There were no withdrawals in the experimental group; however, one participant in the control group was discharged from the facility during the study period, resulting in a total of 23 participants in the experimental group and 22 participants in the control group.

All participants of this study were out-of-school female adolescents who had dropped out or were expelled from school due to running away from home or delinquency and were admitted to the facility under a supervision probation per the Juvenile Act of the Republic of South Korea more than six months prior the study.

### 2.3. Instruments

#### 2.3.1. Alcohol-Related Knowledge

Alcohol-related knowledge was measured using 25 items, consisting of 15 items for alcohol-related knowledge for adolescents applied to female adolescents by Shin [17] and 10 items for knowledge about alcohol during pregnancy developed by Kim et al. [18]. The total score ranged from 0 to 25, with a higher score indicating greater alcohol-related knowledge and awareness of the association between pregnancy and drinking. The reliability (Kuder-Richardson formula 20; KR-20) of the tool was 0.77 in the study by Shin (2006) and 0.76 in this study.

#### 2.3.2. Alcohol Outcome Expectancy

Alcohol outcome expectancy was measured using the 17 items Alcohol Expectancy Scale adapted by Yoon et al. [19] from the 90 items Alcohol Expectancy Questionnaire-Adolescents developed by Christiansen et al. [20] after obtaining permission from the developer. The tool consisted of 17 items in four subscales: sociability, aggressiveness, strengthened sexual functioning, and reduced tension. All items were answered with either yes or no, and the total score ranged from 0 to 17, with a higher score indicating expectations of more positive outcomes of alcohol. The KR-20 was 0.79 for improved sociability, 0.71 for aggression, 0.74 for strengthened sexual functioning, and 0.79 for reduced tension in the study by Yoon et al. [19], and the reliability of the entire tool was 0.87.

#### 2.3.3. Drinking Refusal Self-Efficacy

The Drinking Refusal Self-Efficacy Scale adapted by Cho [21] from the 7 items Adolescents’ self-efficacy for drinking scale developed by Aas et al. [22] was used for this study. Each item was rated on a 5 point Likert scale, and the total score ranged from 7 to 35, with a higher score indicating higher drinking refusal self-efficacy. The reliability of the tool (Cronbach’s α) was 0.86 in the study by Cho [21] and 0.89 in this study.

#### 2.3.4. Alcohol Abstinence Intention

The Alcohol Abstinence Intention Scale was used with permission from its developer, Kim [23], who developed it for female middle school students. An item on alcohol abstinence intention, “I will quit drinking when I plan to conceive,” was added for four items. Each item was rated on a 5 point Likert scale, ranging from strongly disagree (1) to strongly agree (5), and the total score ranged from 4 to 20, with a higher score indicating greater alcohol abstinence intention. The reliability of the tool (Cronbach’s α) was 0.93 in Kim’s [23] study and 0.92 in this study.

### 2.4. Procedure and Data Collection

#### 2.4.1. Program Composition and Contents

The alcohol-related harm prevention program used in this study was designed based on the school-based adolescent drinking prevention program by Yoon [24] and peer leader fostering program to prevent drinking problems in adolescents [10], with the addition of problems caused by drinking in female run-away and delinquent adolescents and other relevant circumstances. The program was designed with advice from an addiction professional.

Our alcohol-related harm prevention program taught adolescents who had already initiated alcohol use or were at high risk for alcohol initiation with frequent exposure to drinking situations about the harm of alcohol consumption. The goal of the program was to help past alcohol users continue to abstain from alcohol or delay relapse after discharge from the facility and to help naïve adolescents refrain from alcohol initiation. The program consisted of six sessions, with one-hour sessions twice a week, and involved group lectures and small group activities.

The participants were given an opportunity to learn about drinking-related problems in females and recognize their drinking tendencies. Furthermore, they were trained on communication skills for drinking situations and practiced role-plays. During the program, the instructor empathized and built a bond of trust with the participants. The participants were instructed to explore the gap between their desired life and current behaviors, and their occasional resistance was accepted. Moreover, it also provided them with an opportunity to think about the pros and cons of drinking alcohol. Motivational interviewing (MI) was attempted, with an attitude encouraging autonomous motivation for behavioral change and abstinence. For reference, the researchers who participated in this study received MI training from a Korean MI expert.

In terms of delivering knowledge, the topics of the group lectures were classified into female adolescents and alcohol, alcohol and stress, and alcohol and communication to explain the ingredients and properties of alcohol, reasons for regulating alcohol consumption in adolescents, and their current status. The topics were also classified into groups such as adolescents’ motives for drinking, the impact of drinking, physiological differences between females and males, developmental features in adolescents, alcohol metabolism and complementary mechanisms of addiction, popular myths about alcohol, emotions that trigger alcohol consumption, stress management, and communication skills. Further, with a focus on running away and delinquency, the social risks of drinking by female adolescents, such as potential sexual violence, violence, date rape, depression and suicide, and fetal alcohol syndrome caused by drinking during pregnancy, were also emphasized. The information was delivered via pictures and videos, along with a virtual drinking experience, an explanation using a human body model, and true-or-false quizzes.

To help participants recognize their drinking tendencies and impart situation-handling skills, their drinking attitudes and alcohol abstinence intention were expressed as mind maps. Activities such as applying cognitive and behavioral techniques to deal with emotions and stressors related to alcohol cravings, finding activities to replace alcohol, relaxing to manage stress, and training on communication, and participating in role-playing to decline drinking invitations were conducted.

To encourage voluntary motivation and improve self-efficacy, the participants were asked to share their positive and negative experiences of drinking alcohol and compare their desired lives 10 years from now with the lives they will have if they continue drinking. They were then instructed to make resolutions and plan specific changes they wanted to make. Additionally, the participants assessed the gains and losses from drinking, identified obstacles that hindered change, and recalled past experiences of achieving behavioral changes to reinforce their positive aspects. The participants also created promotional posters about preventing harm from alcohol based on what they had learned from the program and presented them to one another.

The participants were informed that participation in the program was an opportunity for self-improvement, during which they would be given essential resources to help them make autonomous decisions in drinking situations in the future. Each step of the program was symbolized by the following names: Step 1—seed stage (sessions 1, 2), Step 2—sprouts stage (sessions 3, 4), and Step 3—hope tree stage (sessions 5, 6). The participants were guided to write their resolutions and practical efforts made during the program as well as their dreams on a fruit-shaped figure and hang them up in a tree to inspire them toward change. The first half of the stages consisted of group lectures to deliver information, while the latter half of the stages consisted of three small-group activities with 6–7 people in each group (Table 2).

#### 2.4.2. Data Collection

For participants’ ethical protection, permission to collect data was obtained from the facility, and the study was approved by the institutional review board at Seoul National University (IRB No.1406/001-008). Since the participants lived in the same facility, data were collected from the control group first to prevent the diffusion of the experimental treatment. A baseline survey was conducted using a structured questionnaire regarding the general characteristics and dependent variables, which took about 15 min to complete. As the experimental treatment, the six-session alcohol-related harm prevention program was conducted for the experimental group, with one-hour sessions twice a week. A post-intervention survey was conducted at the final session of the program for the experimental group and at three weeks after the baseline survey for the control group. The post-intervention survey was the same as the baseline survey, except for items relating to general characteristics.

### 2.5. Data Analysis

The collected data were analyzed using the SPSS 26.0 software (IBM, Chicago, IL, USA). The participants’ general characteristics were analyzed with frequency and percentage, and the homogeneity between the experimental and control groups was tested using *t*-test, χ^2^-test, and Fisher’s exact test. The differences between the two groups were analyzed using the analysis of covariance (ANCOVA). The variable that had been heterogeneous between the groups at baseline (alcohol-related knowledge) was analyzed after treating it as a covariate. The reliability of the tools was tested using KR-20 and Cronbach’s α. The significance level of all statistics was set at *p* < 0.05 to accept the hypotheses.

## 3. Results

### 3.1. Participants’ General Characteristics and Homogeneity Testing

The mean age of the participants was 16.02 ± 1.76 years, and all 45 (100%) participants were not attending school (Table 3). The mean age and grade of the experimental group and the control group were 16 years old and 7th to 8th grade. The average age of first drinking was 12 years, and the frequency of drinking was 13 times a month. The average amount of alcohol consumed by the participants was 24 drinks (soju) a day, and the average binge drinking was ten times a month.

The school years at which the participants stopped attending school were 7th grade (n = 7, 15.6%), 8th grade (n = 8, 17.8%), 9th grade (n = 16, 35.6%), 10th grade (n = 10, 22.2%), 11th grade (n = 1, 2.2%), and 12th grade (n = 3, 6.7%). All 45 (100%) students stated that they had consumed alcohol in the past. The mean age of alcohol initiation was 12.91 ± 1.73 years (range 7–17 years). Of the total, 32 (71.1%) had participated in a drinking prevention program in the past, while 13 (28.9%) had not. Drinking prevention education was provided at school (n = 14, 46.7%), home (n = 1, 3.3%), counseling centers after dropping out of school (n = 9, 30.0%), and other facilities. It was also provided by strangers or older schoolmates (n = 6, 20.2%). Before admission to the center, the mean number of days of alcohol use per month was 13.69 ± 10.06 days (range 0–30 days) among the alcohol users, and the mean number of drinks per seating was 24.20 ± 16.19 soju shots (range 0–65 soju shots). Furthermore, the mean number of days of binge drinking (≥ 3 soju shots) per month was 10.69 ± 9.09 days (range 0–30 days). The baseline homogeneity testing showed that there were no statistically significant differences between the experimental and control groups.

Before the alcohol-related harm prevention program, the experimental and control groups did not show a statistically significant difference in alcohol outcome expectancy, drinking refusal self-efficacy, or alcohol abstinence intention. However, they did show a significant difference in alcohol-related knowledge. The mean alcohol-related knowledge score was 13.90 ± 2.05 for the control group and 9.47 ± 3.14 for the experimental group, showing a significantly higher alcohol-related knowledge score (t = 3.53, *p* = 0.018) in the control group (Table 4).

### 3.2. Effects of the Alcohol-Related Harm Prevention Program

#### 3.2.1. Hypothesis 1

ANCOVA was performed to test Hypothesis 1: The experimental group that participated in an alcohol-related harm prevention program would show a greater increase in their alcohol-related knowledge scores compared to the control group. The two groups showed a statistically significant difference in alcohol-related knowledge (F = 1.43, *p* = 0.037) after the intervention; thus, Hypothesis 1 was supported (Table 5).

#### 3.2.2. Hypothesis 2

ANCOVA was performed to test Hypothesis 2: The experimental group that participated in an alcohol-related harm prevention program would show a greater drop in their alcohol outcome expectancy scores compared to the control group. The two groups showed statistically significant differences in alcohol expectancy (F = 2.09, *p* = 0.040) after the intervention; thus, Hypothesis 2 was supported (Table 5).

#### 3.2.3. Hypothesis 3

ANCOVA was performed to test Hypothesis 3: The experimental group that participated in an alcohol-related harm prevention program would show a greater increase in their drinking refusal self-efficacy scores compared to the control group. The two groups did not show statistically significant differences in drinking refusal self-efficacy after the intervention; thus, Hypothesis 3 was rejected (F = 2.90, *p* = 0.966; Table 5).

#### 3.2.4. Hypothesis 4

ANCOVA was performed to test Hypothesis 4: The experimental group that participated in an alcohol-related harm prevention program would show a greater increase in their alcohol abstinence intention scores compared to the control group. The two groups did not show statistically significant differences in abstinence intention after the intervention; thus, Hypothesis 4 was rejected (F = −3.68, *p* = 0.509; Table 5).

## 4. Discussion

This study aimed to assess the effects of an alcohol-related harm prevention program for out-of-school female adolescents who resided in a protection and treatment facility. All of the participants in this study had a history of drinking (100%), which was a markedly higher rate of drinking than that reported by out-of-school youth (83.1%) as per the National Youth Policy Institute [2]. One participant claimed to have begun drinking alcohol by simply trying it out of curiosity at the age of seven years (in 2nd grade), highlighting the seriousness of the dangers of drinking among out-of-school female adolescents who have run away from home and engaged in delinquent behaviors. The mean number of days of drinking per month (13 days) and the mean number of drinks per seating (24 soju shots) also exceeded the criteria for moderate drinking among adults, showing that the intensity of alcohol use was severe. The participants felt an overwhelming alcohol craving after admission to the center and suffered from withdrawal symptoms, such as restlessness and hand tremors, so they also needed therapeutic interventions for alcohol abuse in addition to drinking prevention interventions.

In this study, the experimental group showed an increased alcohol-related knowledge score (*p* = 0.037) and a reduced alcohol outcome expectancy score (*p* = 0.046). This change was significantly greater than that of the control group, which showed that the alcohol-related harm prevention program improved alcohol-related knowledge and converted individuals’ positive expectations of alcohol to negative ones. This result was consistent with previous findings that demonstrated that a drinking prevention program significantly improved alcohol-related knowledge in female middle school students and female vocational high school students [25] and that such programs significantly transformed positive expectations of drinking into negative ones [10,26]. Another study identified alcohol-related knowledge as a predictor of problematic drinking in middle school students [27] and suggested that alcohol-related health education was essential to address the lack of knowledge and information or distorted perceptions and attitudes. However, the participants of our study mentioned that they had already begun using alcohol and had been taught about alcohol from a shelter or adolescent counseling centers after running away from home or from an older schoolmate. This suggested that existing drinking prevention education programs should be expanded to target younger children and adolescents. The marked improvement of the knowledge score in the experimental group in this study showed that repeated education tailored to participants’ interests, level of understanding, and practical problems could provide an intervention to prevent alcohol-related harm. Moreover, these results also supported the IMB model, showing that accurate and appropriate information could transform attitudes and expectations of the outcomes of behavior [28].

In this study, there were no statistically significant changes in drinking refusal, self-efficacy, or alcohol abstinence intention scores in the experimental group after the intervention. Although we included role-play, a strategy validated to increase self-efficacy in previous studies [10], as communication training to refuse to drink, and attempted to motivate participants to change their behaviors by encouraging communication, these were not effective in improving drinking refusal self-efficacy and alcohol abstinence intention. This may be because most prior studies targeted primary prevention to alleviate the individual and social risk factors of drinking and strengthen the protective factors to delay alcohol initiation among in-school adolescents who had not yet begun drinking or had only engaged in moderate drinking [10,11]. However, the participants of this study had already progressed to severe drinking and thus needed therapeutic interventions, as opposed to preventive interventions, with more intensive contents and time. Further, there was a large range of drinking behaviors with varying drinking motives across the participants. Therefore, subsequent studies should design more individualized and intensive interventions as opposed to the group intervention used in this study. Moreover, considering the nature of adolescents, who value a sense of belonging and intimacy and share mutual influences [29,30], it would not be easy for out-of-school female adolescents, who were outside the protective environment of family and school, to abstain from drinking while giving up their needs to belong to a peer group. In this context, despite showing changes in their alcohol-related knowledge and attitude toward drinking, the lack of significant changes in the variables that actually reflected a shift in their behaviors, namely drinking refusal self-efficacy and alcohol abstinence intention, could be viewed as an honest confession of the participants of this study. Furthermore, delinquent adolescents who drank alcohol generally had unsatisfactory bonding with their parents at home, and thus, naturally spent more time with their peers, thereby being heavily influenced by their peer groups [31]. Thus, subsequent studies should also help out-of-school female adolescents recover their relationships with their parents to build a strong bond, and thus, maintain appropriate peer relationships while implementing a strategy of providing education for the entire peer group to transform it into one that had a positive influence. Moreover, studies should also consider employing strategies to form new self-help groups with participants who decided to quit drinking so that they could experience positive peer relations in the group and serve as a teaching model for one another to support change.

In terms of the contents of the program, group lectures and small group activities helped engage the participants by providing an opportunity to explore their drinking habits and motives, discuss alternatives to drinking, and receive feedback from peers who had the same experience or empathize with one another. Further, the dynamics of group activities motivated the participants to change. Specifically, role-playing in the group helped the participants to not only learn communication skills but also observe the drinking behaviors of other people, thereby helping them objectively recognize their own drinking behaviors. The participants had difficulty with cognitive approaches to their emotions and stress but actively engaged in expressing their emotions through behaviors in the role-play. During the cognitive exploration of situations that triggered negative emotions or stress, the participants discovered that their underlying thoughts or beliefs were complicated; thus, leading them to easily give up. They were easily emotionally swayed, even by imagining an unpleasant situation, which hindered objectification. This seemed to be related to the low intellectual level and lack of impulse control among out-of-school youth and the thrill-seeking tendency among people who demonstrated problematic drinking [29,32]. Hence, the participants seemed to have preferred role-plays that involved immediate sharing of emotional responses due to their tendency to seek a powerful experience but having difficulties with cognitive approaches that were time-consuming and required deeper thinking because it felt like studying. This was one reason facilitators of a program should emotionally bond with the participants when proceeding with the program.

As the participants stated that hearing about the harms caused by alcohol in their families or from friends during role-play was a sort of awakening, vicarious experiences would help motivate them and increase their self-efficacy. Subsequent studies may add activities such as listening to the experiences or watching videos of women who recovered from alcoholism or mothers of children suffering from fetal alcohol syndrome to provide an opportunity for participants to vicariously experience the harm of alcohol. Each session of our program lasted for about two hours, so it was difficult for the participants to stay focused throughout the session, as they were not used to sitting for prolonged periods after dropping out of school. Considering past findings that adolescents showing delinquency and drinking alcohol were highly impulsive and had low self-control [33], future studies should consider shortening the duration of each session, adding breaks between the sessions, or adding activities that involve physical movement. Before explaining the perils of alcohol consumption, we expressed respect to our participants by building shared recognition of the fact that they were meaningful solely by their beings. They should value their beautiful bodies and minds as women, and they would be mothers to healthy babies someday. Further, even while providing an educational intervention, we used communication strategies that would motivate participants by presenting specific cases of psychological situations faced by out-of-school youth, and the physical, mental, and social problems caused by drinking, and also sharing our own experiences to build a rapport. We listened to their questions carefully, sometimes answering them immediately, and encouraged them to find their own answers through reflective listening.

One month after the program, five volunteer participants were interviewed. They considered fetal alcohol syndrome to be a memorable topic. All five participants stated that they would not drink alcohol when pregnant or when trying to conceive and that, even if they drink alcohol without knowing they are pregnant, they will stop drinking immediately as soon as they discover their pregnancy. In this study, the participants showed a change in their knowledge and attitude but not in their drinking refusal self-efficacy or alcohol abstinence intentions. When asked about the reason, two participants stated that they had nothing else to do other than drinking when they met up with friends at night, while three participants stated that they could refuse drinking invitations from friends but could not turn down drinking invites from bosses or co-workers for fear of potential disadvantages. Thus, instead of simply urging the individuals to make an effort, social and environmental efforts should be considered to mitigate drinking problems among out-of-school female adolescents.

## 5. Conclusions

The alcohol-related harm prevention program provided in this study was significant as it was a community-based psychiatric intervention program provided to out-of-school youth, who were excluded from the public benefits offered in school. Moreover, the program specifically reflected the unique drinking situations faced by out-of-school female adolescents to render a participant-centered intervention. The increasing out-of-school youth population highlights the need to develop various interventions that promote their health, health management, and self-care abilities. The role of psychiatric nurses is particularly important in this regard as they are the ones who can implement these interventions in place of schools. Thus, it is important to prevent this population group from developing alcoholism and experiencing its harmful effects. For this reason, the intervention program of this study was important in the field of addiction.

This study has several limitations. It was conducted on female adolescents admitted to a corrective facility; thus, the results should be generalized to the overall female adolescent population or all out-of-school adolescents with caution. Further, we only examined the effects of the alcohol-related harm prevention program using a pretest–post-test design, so continuous observation would be needed to substantiate the continuous effects of the intervention.

## Figures and Tables

**Table 1:** Research design.

Group	Pretest	Post-Test	Pretest	Intervention	Post-Test
Control group	C1	C2			
Experimental group			E1	X	E2

C1, E1 = general characteristics, alcohol-related knowledge, alcohol outcome expectancy, drinking refusal self-efficacy, alcohol abstinence intention; C2, E2 = alcohol-related knowledge, alcohol outcome expectancy, drinking refusal self-efficacy, alcohol abstinence intention; X = alcohol-related harm prevention program.

**Table 2:** Sessions and contents of the alcohol-related harm prevention program.

State	Session	Contents	Time (min)
1 Seed stage	1	Orientation Lecture and Discussion: Female adolescents and alcohol consumption - Alcohol and its metabolism - Alcohol consumption in Korean adolescents - The physiological difference between woman and man, and adolescent and adult - Alcohol-related harm	50
	2	Small group activity - Drawing a mind-map of alcohol consumption - Sharing experiences of alcohol-related harm Wrap up	50
2 Sprout stage	3	Orientation: Quiz Lecture and Discussion: Stress and alcohol consumption - Motivation for drinking in adolescents - Controlling alcohol-related emotions and stress management - Effect of mass media	50
	4	Small group activity - Cognitive approach - Relaxation training (abdominal respiration, progressive muscle relaxation) Wrap up	50
3 Hope-tree stage	5	Orientation Lecture and Discussion: Alcohol consumption and communication - O/X Quiz - Autonomous decision-making and communication skills	50
	6	Small group activity - Expressing refusal through role play - Drawing an alcohol prevention poster - Making a hope-tree (My dream, what I am, commitment) Wrap up (10 min): Awards ceremony and post-test	50

**Table 3:** General characteristics of the participants.

Characteristics	Categories	Exp. (n = 23) n (%) Mean ± SD	Con. (n= 22) n (%) Mean ± SD	Total (n = 45) n (%) Mean ± SD	χ^2^ or *t*	*p*
Age (years)		16.00 ± 1.90	16.05 ± 1.64	16.02 ± 1.76	0.08	0.932
Education	Middle school 1	4(17.4)	3(13.6)	7(15.6)	7.70	0.135
	Middle school 2	5(21.7)	3(13.6)	8(17.8)		
	Middle school 3	5(21.7)	11(50.0)	16(35.6)		
	High school 1	8(34.8)	2(9.1)	10(22.2)		
	High school 2	0(0.0)	1(4.5)	1(2.2)		
	High school 3	1(4.3)	2(9.1)	3(6.7)		
First Drinking (years)		12.78 ± 2.13	13.05 ± 1.21	12.91 ± 1.73	0.50	0.616
Drinking Frequency (days/month)		13.30 ± 8.99	14.09 ± 11.27	13.69 ± 10.06	0.25	0.797
Drinking Amount (standard drinks/day)		24.00 ± 13.55	24.41 ± 18.88	24.20 ± 16.19	0.08	0.934
Binge Drinking (days/month)		10.65 ± 8.64	10.73 ± 9.74	10.69 ± 9.09	0.02	0.978

**Table 4:** Homogeneity of outcome variables (*N* = 45).

Variables	Exp. (n = 23)	Cont. (n = 22)	*t*	*p*
	Mean ± SD			
Alcohol-related knowledge	9.47 ± 3.14	13.90 ± 2.05	3.53	0.018
Alcohol expectancy	10.13 ± 3.26	10.09 ± 2.92	−0.04	0.966
Drinking refusal self-efficacy	22.61 ± 8.47	22.86 ± 6.90	0.11	0.913
Abstinence intention	14.30 ± 3.81	14.05 ± 4.30	−0.21	0.832

**Table 5:** Comparison of the differences of dependent variables between groups (*N* = 45).

Variable	Time	Exp. (n = 23)	Cont. (n = 22)	*t* or F	*p*
		Mean ± SD			
Alcohol-related knowledge	Pre	9.47 ± 3.14	13.90 ± 2.05		
	Post	20.81 ± 11.35	14.32 ± 2.39	1.43	0.037
Alcohol expectancy	Pre	10.13 ± 3.26	10.09 ± 2.92		
	Post	7.96 ± 4.45	9.95 ± 3.18	2.09	0.046
Drinking refusal self-efficacy	Pre	22.61 ± 8.47	22.86 ± 6.90		
	Post	24.78 ± 7.61	23.68 ± 6.44	2.90	0.966
Abstinence intention	Pre	14.30 ± 3.81	14.05 ± 4.30		
	Post	15.61 ± 3.73	14.73 ± 3.80	3.68	0.509

## Data Availability

No new data were created or analyzed in this study. Data sharing is not applicable to this article.
